# The Gluco-Vascular Injury Axis in Diabetic Cardiovascular Dysfunction: A Narrative Review

**DOI:** 10.7759/cureus.110771

**Published:** 2026-06-13

**Authors:** Mohammad Faraz Omair, Hrishikesh Bhalchandra Desle, Srinidhi S Hegde, Vaishali K Chavan, Yagnik Prafulchandra Tank, Himanshu Jha

**Affiliations:** 1 Department of Internal Medicine, St. Peter’s Hospital, Chertsey, GBR; 2 Department of Cardiology, Heart and Soul Super Speciality Hospital, Malegaon, IND; 3 Department of Cardiology, Sri Jayadeva Institute of Cardiovascular Sciences and Research, Mysuru, IND; 4 Department of Pharmacy, Anjuman-I-Islam's Kalsekar Technical Campus (AIKTC), Navi Mumbai, IND; 5 Department of Pathology, Dr. N. D. Desai Faculty of Medical Science and Research, Dharmsinh Desai University, Nadiad, IND; 6 Department of Family Medicine, Down Town Hospital, Guwahati, IND

**Keywords:** arterial stiffness, diabetic cardiomyopathy, endothelial dysfunction, gluco-vascular injury axis, oxidative stress

## Abstract

Diabetic cardiovascular dysfunction arises from a progressive metabolic-vascular-myocardial injury pathway rather than from hyperglycemia alone. In this review, the “gluco-vascular injury axis” is used as a proposed conceptual framework describing how diabetes-related metabolic stress promotes endothelial dysfunction, vascular injury, microvascular impairment, myocardial remodeling, and heart failure. Chronic hyperglycemia, insulin resistance, dyslipidemia, oxidative stress, inflammation, AGE-RAGE (advanced glycation end products-receptor for advanced glycation end products) signaling, mitochondrial impairment, arterial stiffness, and coronary microvascular dysfunction contribute to fibrosis, diastolic dysfunction, and heart failure. Because these mechanisms are often discussed separately, the primary clinical message can become obscured. This narrative review synthesizes mechanistic, experimental, and clinical evidence to organize these processes into a clinically interpretable continuum. The novelty of this review lies in integrating diabetic cardiomyopathy, atherosclerosis, endothelial dysfunction, and coronary microvascular disease into one metabolic-vascular-myocardial framework, with endothelial dysfunction positioned as the transition point between glucose-lipid stress and cardiovascular injury. It was not designed as a systematic review and did not use formal search, screening, or meta-analytic methods. The review highlights metabolic triggers, endothelial injury, inflammatory amplification, macrovascular disease, microvascular dysfunction, mitochondrial redox imbalance, and diabetic cardiomyopathy. The key takeaway is that cardiovascular protection in diabetes may need to address the entire gluco-vascular injury process through a combination of metabolic, endothelial, inflammatory, mitochondrial, microvascular, and myocardial protective strategies.

## Introduction and background

Diabetes mellitus is now understood as a systemic cardiometabolic disease rather than only a disorder of chronic hyperglycemia [1]. Its cardiovascular burden reflects the combined effects of glucose excess, insulin resistance, dyslipidemia, inflammation, oxidative stress, endothelial dysfunction, and maladaptive myocardial remodeling [2]. Diabetic patients are therefore at higher risk for coronary artery disease, heart failure, peripheral artery disease, microvascular disease, and cardiovascular mortality [1]. These complications should not be viewed as isolated outcomes. They represent a progressive injury pathway in which metabolic stress first damages the vasculature and then contributes to myocardial dysfunction [3]. Endothelial dysfunction is a central link in this pathway because the endothelium regulates vascular tone, nitric oxide bioavailability, thrombosis, vascular permeability, inflammation, and vascular repair [4]. Diabetic metabolic stress reduces nitric oxide signaling, increases reactive oxygen species (ROS) generation, activates inflammatory pathways, and shifts the vascular wall toward a pro-adhesive and pro-thrombotic phenotype [5]. In this review, the term “gluco-vascular injury axis” is used as a proposed conceptual framework rather than as an established diagnostic entity. It refers to the continuum linking diabetes-related metabolic stress with endothelial dysfunction, macrovascular and microvascular injury, impaired myocardial perfusion, myocardial fibrosis, diastolic dysfunction, and heart failure.

Oxidative stress provides an early mechanism through which metabolic injury becomes vascular injury. Hyperglycemia and insulin resistance promote mitochondrial dysfunction, nicotinamide adenine dinucleotide phosphate (NADPH) oxidase activation, antioxidant depletion, and endothelial nitric oxide synthase (eNOS) uncoupling [6]. These changes impair vascular relaxation, increase endothelial permeability, promote leukocyte adhesion, and contribute to vascular remodelling [3]. Clinical data also suggest that poor glycemic control in type 2 diabetes is associated with endothelial dysfunction and coronary artery disease [7]. After endothelial injury is established, diabetic vascular disease progresses through both macrovascular and microvascular pathways. Macrovascular disease includes accelerated atherosclerosis, coronary artery disease, peripheral artery disease, arterial stiffness, vascular calcification, and plaque instability [1,3]. Inflammation, extracellular matrix remodeling, adipose tissue dysfunction, and epicardial adipose tissue may further contribute to arterial stiffness in type 2 diabetes and cardiovascular disease [8].

Microvascular injury provides the clinical bridge between systemic metabolic stress and impaired tissue perfusion. Diabetic microvascular disease involves endothelial injury, capillary loss, basement membrane thickening, pericyte dysfunction, reduced vasodilatory reserve, and impaired tissue perfusion [8]. Coronary microvascular dysfunction can limit myocardial oxygen delivery even without obstructive epicardial coronary stenosis [9]. This point is clinically important because diabetic patients may develop myocardial dysfunction despite the absence of flow-limiting coronary artery disease. Diabetic cardiomyopathy, therefore, reflects more than intrinsic cardiomyocyte dysfunction; it emerges from metabolic, endothelial, inflammatory, and microvascular injury pathways [10].

At the myocardial level, this vascular injury pathway promotes energetic failure, fibrosis, and impaired relaxation. Diabetes adversely affects mitochondrial function, oxidative balance, lipid handling, advanced glycation, calcium cycling, cardiomyocyte stress, inflammation, and interstitial fibrosis [2]. Endothelial dysfunction contributes to this process by disrupting coronary microvascular perfusion, reducing nitric oxide-mediated cardiomyocyte signaling, increasing vascular inflammation, and promoting myocardial fibrosis [10]. These mechanisms lead to impaired relaxation, diastolic dysfunction, and heart failure [11]. Diabetes also interacts with renal dysfunction and heart failure through fluid retention, neurohormonal activation, inflammation, endothelial injury, and systemic vascular dysfunction [12]. The diabetes-heart failure relationship is therefore best interpreted as a shared metabolic, vascular, renal, and myocardial injury network rather than as a coincidental association [11].

Inflammation amplifies this network and helps sustain the transition from metabolic stress to cardiovascular damage. Inflammasomes, including NLRP3, connect metabolic stress, endothelial inflammation, cytokine production, vascular dysfunction, and cardiovascular injury in type 2 diabetes [13]. Diabetic inflammatory activation also increases endothelial adhesion molecule expression, inflammatory cell recruitment, oxidative injury, and vascular smooth muscle dysfunction [3]. In heart failure, disrupted interactions between endothelial cells, cardiomyocytes, and immune cells further contribute to myocardial and endothelial dysfunction [14]. These findings support endothelial injury as a dynamic pathological node rather than a passive consequence of hyperglycemia.

The gluco-vascular injury axis offers a unifying narrative for these mechanisms. Although existing reviews have described diabetic cardiomyopathy, endothelial dysfunction, atherosclerosis, inflammation, oxidative stress, and coronary microvascular dysfunction in detail, these processes are often presented as separate or parallel complications. This creates a knowledge gap: the field lacks a simple integrative framework that explains how diabetic metabolic stress is progressively translated into vascular injury and then myocardial dysfunction. In schematic terms, the proposed axis follows four linked stages: metabolic stress, including hyperglycemia, glycemic variability, insulin resistance, and dyslipidemia; endothelial transition, marked by nitric oxide depletion, oxidative stress, glycocalyx injury, barrier dysfunction, and inflammation; vascular amplification, involving macrovascular remodeling, arterial stiffness, coronary microvascular dysfunction, impaired perfusion, and tissue hypoxia; and myocardial expression, characterized by mitochondrial dysfunction, fibrosis, diastolic dysfunction, diabetic cardiomyopathy, and heart failure. This axis describes a self-perpetuating sequence in which chronic metabolic stress causes endothelial and vascular injury, impaired vascular signaling, inflammatory amplification, myocardial fibrosis, and cardiac dysfunction [10,15,16].

The central message is that vascular injury is the mechanism through which metabolic dysregulation is translated into cardiovascular dysfunction. A new unifying framework is needed because glycemic burden alone does not fully explain the heterogeneity, timing, or progression of cardiovascular complications in diabetes. Unlike conventional diabetic cardiomyopathy models that primarily emphasize direct cardiomyocyte injury, altered substrate metabolism, mitochondrial dysfunction, calcium-handling abnormalities, lipotoxicity, and fibrosis, this framework positions endothelial dysfunction and vascular impairment as the active mechanistic bridge between systemic metabolic stress and myocardial remodeling. This framework integrates altered glycemic status, endothelial dysfunction, coronary microvascular disease, and diabetic cardiomyopathy into a single clinically relevant pathway [3,4,8,10]. Its mechanistic distinction is that vascular injury is treated not as a parallel complication, but as an intermediary stage that helps explain ischemia, fibrosis, and heart failure even without obstructive epicardial coronary artery disease. A gluco-vascular approach may improve early risk stratification, support the discovery of vascular biomarkers, and guide therapies targeting glucose toxicity, endothelial dysfunction, oxidative stress, inflammation, microvascular dysfunction, and myocardial remodeling [2,8,10,11].

Objective of the review

This narrative review integrates mechanistic, experimental, and clinical evidence on the gluco-vascular injury axis in diabetic cardiovascular dysfunction. It was not designed as a systematic review and did not use formal systematic search, screening, or meta-analytic methods. No meta-analysis, meta-regression, pooled effect estimate, confidence interval calculation, heterogeneity analysis, or P-value-based statistical comparison was planned or performed because the review was designed as a narrative synthesis. To improve transparency, relevant literature was identified through a narrative search of PubMed/MEDLINE, Scopus, Google Scholar, and reference lists of relevant articles, prioritizing peer-reviewed studies and reviews addressing diabetic cardiovascular disease, endothelial dysfunction, diabetic cardiomyopathy, coronary microvascular dysfunction, oxidative stress, inflammation, AGE-RAGE (advanced glycation end products-receptor for advanced glycation end products) signaling, mitochondrial injury, arterial stiffness, and diabetes-associated heart failure. Articles were selected based on relevance to mechanistic, experimental, and clinical aspects of the proposed metabolic-vascular-myocardial framework rather than by predefined systematic eligibility criteria. Because no predefined systematic search protocol, duplicate screening, formal risk-of-bias assessment, or meta-analysis was used, selection bias cannot be excluded.

The objective is to address the current fragmentation in the literature by organizing metabolic, endothelial, inflammatory, mitochondrial, microvascular, and myocardial mechanisms into a single explanatory framework. Specifically, the review presents a clinically coherent framework in which hyperglycemia, insulin resistance, endothelial dysfunction, oxidative stress, inflammation, mitochondrial injury, and microvascular abnormalities converge to promote diabetic cardiomyopathy and heart failure. This framework is intended to advance current understanding by clarifying the sequence through which metabolic stress becomes vascular dysfunction and then cardiac dysfunction, rather than treating these abnormalities as isolated disease components. The intended takeaway is that cardiovascular protection in diabetes requires more than glycemic control alone; it also requires attention to endothelial, inflammatory, mitochondrial, microvascular, and myocardial injury pathways.

## Review

Conceptual framework: defining the gluco-vascular injury axis

The gluco-vascular injury axis provides a narrative framework for explaining how diabetes-related metabolic stress progresses into vascular and myocardial disease. In this framework, diabetes-induced metabolic dysfunction causes vascular injury, which subsequently promotes cardiovascular dysfunction [15,16]. The proposed axis is not intended to rename diabetic cardiomyopathy or replace established models. Instead, it defines a vascular-centered sequence in which metabolic stress induces endothelial transition, amplifies macrovascular and microvascular injury, and contributes to myocardial remodeling and heart failure.

Schematic definition of the axis includes four linked stages: (1) metabolic stress, including chronic hyperglycemia, glycemic variability, insulin resistance, dyslipidemia, glucotoxicity, and lipotoxicity; (2) endothelial transition, including nitric oxide depletion, oxidative stress, inflammatory activation, glycocalyx injury, barrier dysfunction, and pro-thrombotic signaling; (3) vascular amplification, including arterial stiffness, atherosclerotic remodeling, coronary microvascular dysfunction, impaired perfusion reserve, rarefaction, and tissue hypoxia; and (4) myocardial expression, including mitochondrial energetic failure, cardiomyocyte stress, extracellular matrix remodeling, fibrosis, diastolic dysfunction, diabetic cardiomyopathy, and heart failure. Chronic hyperglycemia increases mitochondrial ROS, redox imbalance, and molecular injury pathways in cardiomyocytes and vascular cells [15]. Recurrent glycemic fluctuations add a second layer of injury by repeatedly exposing vascular tissue to oxidative and inflammatory stress, a process commonly described as glucose variability [16].

This upstream metabolic injury is not limited to glucose excess. Abnormal insulin sensitivity and disordered lipid metabolism promote toxic lipid intermediate accumulation, mitochondrial dysfunction, and impaired substrate use in the myocardium and vasculature [16]. The interaction between glucotoxicity and lipotoxicity is therefore central to the axis because diabetic cardiovascular injury usually reflects combined glucose-lipid stress rather than hyperglycemia alone [16]. This explains why glycemic burden alone may not fully account for cardiovascular heterogeneity in diabetes.

Vascular injury is the main effector stage of the axis. Endothelial dysfunction translates metabolic stress into structural and functional cardiovascular abnormalities [17]. In cardiometabolic disease, endothelial cells lose their ability to regulate nitric oxide-dependent vasodilation, anti-inflammatory signaling, antithrombotic balance, and vascular barrier integrity [17]. Oxidative stress reduces nitric oxide availability and promotes endothelial activation, remodeling, permeability, inflammation, and vasoconstriction [15,17]. Inflammatory activation then amplifies the same process through cytokine release, leukocyte adhesion, endothelial injury, and impaired vascular repair [18]. Experimental evidence showing that reduction of oxidative stress, inflammation, and apoptosis can attenuate diabetic cardiomyopathy further supports the relevance of these pathways [18,19]. This endothelial transition is the mechanistically distinct feature of the proposed axis: vascular injury is treated as an active intermediary between metabolic dysregulation and myocardial damage, not merely as a coexisting diabetic complication.

The downstream clinical consequence is impaired perfusion and myocardial remodeling. Oxidative and inflammatory injury disrupts coronary microvascular regulation, reduces myocardial perfusion reserve, and promotes microvascular rarefaction and tissue hypoxia [17]. Persistent microvascular stress may then cause cardiomyocyte death, extracellular matrix remodeling, myocardial fibrosis, ventricular stiffening, and diastolic dysfunction [19]. This temporal sequence helps explain why diabetic patients may develop myocardial ischemia, impaired relaxation, or heart failure even without obstructive epicardial coronary artery disease.

Compared with conventional diabetic cardiomyopathy models, which often emphasize direct cardiomyocyte injury, altered substrate metabolism, mitochondrial dysfunction, calcium-handling abnormalities, lipotoxicity, autonomic imbalance, renal dysfunction, and fibrosis, the gluco-vascular injury axis places endothelial dysfunction and vascular impairment upstream of myocardial expression. The key clinical takeaway is that diabetic cardiomyopathy and heart failure are the final expression of an interconnected metabolic-vascular-myocardial pathway, not isolated complications of diabetes. The proposed axis begins with glucose toxicity, glycemic variability, insulin resistance, and lipotoxicity; progresses through endothelial dysfunction, nitric oxide depletion, oxidative stress, inflammatory activation, and barrier disruption; and culminates in impaired perfusion, myocardial fibrosis, diastolic dysfunction, and heart failure [15,16,19]. This framework helps convert a descriptive list of mechanisms into a clinically useful sequence that may guide earlier risk recognition and multi-targeted intervention. The main players in the gluco-vascular injury axis are described in Table 1.

**Table 1:** Key Components of the Gluco-Vascular Injury Axis

Component	Main mechanism	Outcome	Reference
Hyperglycemia	Increases mitochondrial reactive oxygen species and redox imbalance	Cardiomyocyte and vascular-cell injury	[15]
Glycemic variability	Triggers repeated oxidative and inflammatory stress	Endothelial and myocardial damage	[16]
Insulin resistance	Disrupts substrate use and metabolic signaling	Vascular dysfunction and myocardial stress	[16]
Lipid dysregulation	Promotes lipotoxicity and disturbed glucose-lipid interaction	Mitochondrial and vascular injury	[16]
Endothelial dysfunction	Reduces nitric oxide, barrier function, and vascular protection	Vascular remodeling and impaired perfusion	[17]
Inflammation	Increases cytokine activity, endothelial injury, and inflammatory signaling	Progressive vascular and cardiac damage	[13,18]
Oxidative stress	Promotes endothelial activation, apoptosis, and myocardial injury	Fibrosis and cardiac dysfunction	[15,18,19]
Microvascular impairment	Reduces coronary regulation and perfusion reserve	Tissue hypoxia and diabetic cardiac dysfunction	[8,17]
Myocardial remodeling	Causes fibrosis, stiffness, and diastolic dysfunction	Diabetic cardiomyopathy and heart failure	[2,10,19]

Metabolic triggers of vascular injury in diabetes

The metabolic trigger layer of the gluco-vascular injury axis begins with sustained glucose and lipid overload. In diabetes, hyperglycemia and hyperlipidemia initiate vascular damage by promoting endothelial dysfunction, inflammation, oxidative stress, and vascular remodeling [20]. This section, therefore, explains the first step in the clinical sequence: how abnormal metabolism becomes vascular injury. Within the proposed axis, these metabolic abnormalities are not independent risk factors; they are upstream triggers that converge on endothelial transition and initiate the vascular phase of diabetic cardiovascular dysfunction.

Intracellular glucose accumulation alters endothelial nitric oxide signaling, reduces endothelial vasodilator capacity, increases vascular permeability, and activates endothelial cells [21]. These mechanisms make chronic hyperglycemia a key upstream event in diabetic macrovascular and microvascular complications [22]. Glucose variability adds further injury because repeated glycemic fluctuations expose endothelial cells to recurrent oxidative and inflammatory stress [23]. Thus, sustained hyperglycemia provides chronic biochemical pressure, whereas glycemic variability provides repeated injury pulses that accelerate endothelial activation, barrier disruption, and loss of nitric oxide bioavailability [21-23].

Insulin resistance then reinforces this vascular injury. Under physiological conditions, insulin supports endothelial nitric oxide production through the PI3K-Akt-eNOS pathway; in insulin resistance, this protective pathway is impaired [23,24]. Compensatory hyperinsulinemia and mitogen-activated signaling may also promote vasoconstriction, smooth muscle proliferation, inflammation, and atherogenesis [23]. In the gluco-vascular injury axis, insulin resistance therefore functions as a signaling switch: it weakens vasodilatory and anti-inflammatory insulin signaling while preserving or amplifying pro-growth and pro-atherogenic pathways [23,24].

Lipid dysregulation acts in parallel with glucose toxicity. Hyperglycemia, lipotoxicity, and dyslipidemia work together to promote vascular damage [20]. Elevated free fatty acids, oxidized low-density lipoprotein (LDL), triglyceride-rich lipoproteins, and toxic lipid intermediates increase oxidative stress, disturb endothelial metabolism, and contribute to plaque formation [23]. The combined effect of glucotoxicity and lipotoxicity, therefore, helps explain the increased vascular risk in type 2 diabetes [20,23]. Lipotoxicity also extends the axis beyond glucose-centered injury by linking excess fatty acid flux, mitochondrial stress, endothelial dysfunction, and inflammatory vascular remodeling [20,23]. A further clinical concern is metabolic memory, in which earlier hyperglycemic injury may continue to influence vascular risk even after later glycemic improvement. Persistent oxidative stress, inflammatory priming, endothelial dysfunction, and epigenetic changes may allow prior metabolic injury to leave a durable vascular imprint [22].

**Figure 1:**
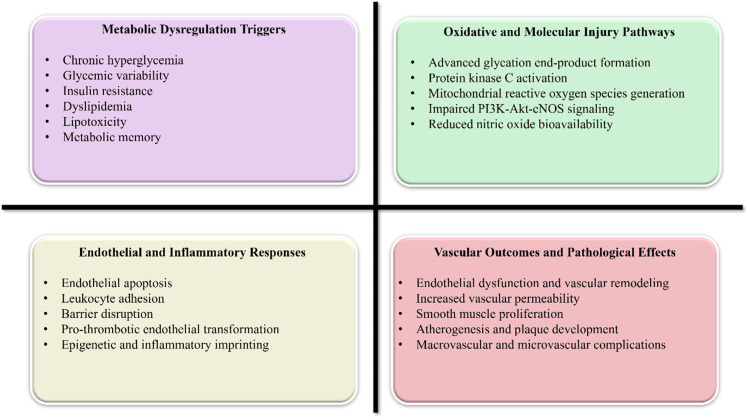
Metabolic Triggers Initiating the Gluco-Vascular Injury Axis in Diabetes Created by the authors using Microsoft PowerPoint (Microsoft® Corp., Redmond, WA).

The metabolic trigger layer consists of hyperglycemia, glycemic variability, insulin resistance, dyslipidaemia, lipotoxicity, and metabolic memory. These factors initiate the gluco-vascular injury axis by converting metabolic overload into endothelial dysfunction and progressive vascular damage [20-23]. This axis-focused interpretation reduces the metabolic trigger layer to a clear sequence: chronic glucose exposure, glucose excursions, impaired insulin signaling, and lipid toxicity converge on oxidative stress, endothelial dysfunction, and vascular remodeling [20-23]. Diabetes can lead to vascular damage through several metabolic pathways, as summarized in Figure 1.

Endothelial dysfunction: the core of the axis

Endothelial dysfunction is the central pathological node of the gluco-vascular injury axis because it converts metabolic stress into impaired vascular regulation, inflammation, thrombosis, tissue hypoperfusion, and myocardial injury [3-6,10]. Under physiological conditions, the endothelium maintains vascular patency, vascular tone, permeability, antithrombotic balance, paracrine signaling, and leukocyte trafficking within vascular and myocardial tissues [3,4]. Hyperglycemia, insulin resistance, dyslipidemia, oxidative stress, and inflammatory signals disrupt this regulatory phenotype in diabetes [3-6,21,22]. The clinical implication is direct: once endothelial function is impaired, metabolic disease becomes a vascular disease.

The first major consequence is loss of nitric oxide-mediated vascular protection. Endothelial dysfunction makes the vascular wall more vasoconstrictive, immune-cell adhesive, permeable, lipid-prone, and maladaptively remodeled [3,25]. Reduced eNOS activity, eNOS uncoupling, decreased tetrahydrobiopterin, increased asymmetric dimethylarginine (ADMA), and oxidative nitric oxide inactivation impair vasodilation [4-6]. Loss of nitric oxide bioavailability also weakens anti-inflammatory and antithrombotic endothelial defenses, increasing leukocyte adhesion, platelet activation, and vascular smooth muscle dysfunction [3,5,6].

Oxidative and nitrosative stress further amplify endothelial injury. In diabetic mice, activation of the AMPK/miR-181b axis has been shown to alleviate endothelial dysfunction and vascular inflammation, suggesting that these pathways may modulate diabetic vascular injury [26]. Increased mitochondrial ROS production damages endothelial proteins, lipids, DNA, and mitochondrial function, while NADPH oxidase activation further increases oxidative injury and peroxynitrite formation [6,26]. These processes suppress nitric oxide signaling, activate inflammatory transcriptional pathways, and promote endothelial apoptosis or senescence [3,6,26].

Structural injury then extends endothelial dysfunction beyond impaired vasodilation. Endothelial injury is associated with impaired cell migration, lipid dysregulation, defective vascular repair, and inflammation [3,21,22]. A key structural component is the degradation of the endothelial glycocalyx. Loss of heparan sulfate, syndecan-1, and hyaluronan damages the endothelial surface layer and increases vascular permeability [3,4,21]. Glycocalyx injury also increases leukocyte adhesion, reduces mechanotransduction and shear-stress sensing, and promotes microvascular leakage [3,4]. Together with endothelial barrier dysfunction, these changes cause vascular leakage, edema, inflammatory cell infiltration, tissue hypoxia, and impaired organ perfusion [3,4,8].

Endothelial dysfunction is not uniform across the vascular system. Coronary, renal, retinal, cerebral, and peripheral endothelial cells differ in metabolic demand, barrier function, inflammatory response, and exposure to local hemodynamic stress [3,4,21,27]. This vascular-bed heterogeneity helps explain the different clinical phenotypes of diabetes, including coronary microvascular dysfunction, nephropathy, retinopathy, cerebrovascular disease, and peripheral vascular disease [3,4,8,21,28]. Circulating biomarkers related to endothelial dysfunction may also serve as markers of systemic vascular injury [3,4].

The key takeaway is that endothelial dysfunction links metabolic overload to reduced blood flow, inflammation, thrombosis, vascular leakage, and cardiovascular dysfunction [3-6,10,21,22]. This makes the endothelium the main biological and clinical bridge between diabetes and downstream macrovascular, microvascular, and myocardial complications. The mechanisms of endothelial dysfunction are outlined in Table 2.

**Table 2:** Endothelial Mechanisms Linking Metabolic Stress to Vascular Injury eNOS: endothelial nitric oxide synthase; miRs: microRNAs; NADPH: nicotinamide adenine dinucleotide phosphate

Component	Main mechanism	Vascular consequence	Reference
Metabolic endothelial injury	Hyperglycemia, insulin resistance, dyslipidemia, oxidative stress, and inflammation damage the endothelial structure and function	Impaired vascular regulation and tissue hypoperfusion	[3-6,21,22]
Nitric oxide impairment	Reduced eNOS activity, eNOS uncoupling, and oxidative nitric oxide inactivation	Defective vasodilation and loss of vascular protection	[4-6]
Pro-inflammatory endothelial shift	Increased leukocyte adhesion, platelet activation, and inflammatory signaling	Vascular inflammation and thrombosis	[3,5,6,13]
Oxidative and nitrosative stress	Mitochondrial reactive oxygen species, NADPH oxidase activation, and peroxynitrite formation	Endothelial apoptosis, senescence, and vascular remodeling	[3,6,26]
Impaired endothelial repair	Altered cell migration, lipid dysregulation, and defective vascular repair	Structural endothelial deterioration	[3,21,22]
Glycocalyx degradation	Loss of heparan sulfate, syndecan-1, and hyaluronan	Increased permeability, leukocyte adhesion, and microvascular leakage	[3,4,21]
Barrier dysfunction	Disrupted endothelial barrier integrity	Edema, inflammatory infiltration, tissue hypoxia, and impaired perfusion	[3,4,8,21]
Vascular-bed heterogeneity	Coronary, renal, retinal, cerebral, and peripheral endothelial cells differ in function and stress response	Diverse diabetic vascular complications	[3,4,8,21,22]
Circulating microRNAs	Altered endothelial dysfunction-related miRs reflect systemic endothelial injury	Potential biomarker role	[3,4]

AGE-RAGE signaling and vascular damage

AGE-RAGE signaling explains how chronic hyperglycemia can leave a durable vascular imprint even after glucose control improves. Advanced glycation end products (AGEs) contribute to diabetic vascular complications by linking chronic hyperglycemia with oxidative stress, inflammation, extracellular matrix remodeling, and endothelial dysfunction [29]. AGEs are produced endogenously through non-enzymatic glycation and oxidation of proteins, lipids, and nucleic acids, particularly during chronic hyperglycemia and increased oxidative stress [29-31]. They may also accumulate through exogenous sources such as smoking and thermally processed foods [31]. In diabetes, persistent AGE accumulation alters vascular protein structure, disrupts cell-matrix and cell-cell signaling, and increases cardiovascular injury [32].

The main inflammatory effect of AGEs occurs through the receptor for advanced glycation end products (RAGE). AGE-RAGE binding activates NF-κB, mitogen-activated protein kinase pathways, oxidative stress, and cytokine production, thereby promoting vascular inflammation [32]. This activation increases adhesion molecule expression, leukocyte recruitment, vascular permeability, and the development of a pro-inflammatory and pro-thrombotic endothelial phenotype [29,32]. RAGE signaling, therefore, reinforces endothelial dysfunction and accelerates diabetic vascular complications [29,32].

AGEs also convert metabolic injury into structural vascular disease through extracellular matrix remodeling. Glycation of long-lived matrix proteins, including collagen and elastin, promotes crosslinking, loss of vascular elasticity, and arterial stiffness [29]. This stiffening reduces vascular compliance, increases pulse-wave velocity (PWV), raises systolic load, and contributes to ventricular afterload [29]. These structural changes provide a direct pathway from chronic hyperglycemia to impaired ventricular relaxation, diastolic dysfunction, and progressive cardiovascular tissue dysfunction [29].

AGE-RAGE signaling further affects vascular smooth muscle cells and macrophages, linking endothelial injury with atherosclerosis and calcification [32]. In vascular smooth muscle cells, this pathway promotes proliferation, migration, oxidative stress, inflammatory activation, phenotypic switching, remodeling, and calcification [32]. In macrophages, AGE-RAGE activation promotes lipid accumulation, foam cell formation, cytokine secretion, impaired efferocytosis, and plaque instability [29]. These cellular effects increase atherosclerotic burden and make diabetic vascular lesions more vulnerable [29].

The therapeutic implication is that AGE-RAGE signaling is a potential target within the gluco-vascular injury axis. Proposed strategies include reducing AGE formation, limiting exogenous AGE intake, inhibiting AGE-RAGE interaction, blocking downstream inflammatory cascades, and enhancing AGE detoxification [29,31,32]. Although clinical translation remains challenging, AGE-RAGE signaling provides a coherent mechanism linking hyperglycemia to endothelial dysfunction, arterial stiffness, atherosclerosis, impaired angiogenesis, and myocardial injury [29,33]. Within the gluco-vascular injury axis, AGE-RAGE signaling should be viewed as a chronic mediator of vascular damage that may persist despite later improvement in metabolic control.

Mitochondrial dysfunction and redox collapse

Mitochondrial dysfunction is a key intracellular link between diabetic metabolic overload and vascular-myocardial injury. In diabetes, excess glucose and lipid substrate delivery increase cellular redox imbalance, oxidative stress, senescence, and cell injury [15,16]. During chronic hyperglycemia and lipid excess, increased substrate flux overloads the mitochondrial electron transport chain and favors superoxide production [15,16]. This mitochondrial ROS burden damages proteins, lipids, mitochondrial DNA, and respiratory complexes, impairing cellular function and vascular homeostasis [6,15,16]. In endothelial cells, mitochondrial oxidative stress reduces nitric oxide bioavailability, activates inflammatory signaling, and promotes oxidative vascular damage [3,6].

Mitochondrial quality control failure then sustains this injury. Cardiovascular injury in diabetes disrupts mitochondrial dynamics, including the balance between fission, fusion, biogenesis, and mitophagy [2,15,16]. Under normal conditions, these processes preserve mitochondrial quality and cellular metabolic stability [15,16]. Disruption of this balance causes excessive mitochondrial fragmentation, impaired fusion, reduced clearance of damaged mitochondria, and further ROS generation [15,16]. These abnormalities promote endothelial senescence, defective vascular repair, inflammatory pathway activation, and progressive microvascular damage [3,6,8]. Thus, impaired mitochondrial quality control links persistent endothelial injury with vascular ageing in diabetes [34].

The endothelial consequence is clinically important because the endothelium regulates vascular tone, permeability, thrombosis, leukocyte adhesion, and tissue perfusion [3,4]. Mitochondrial redox imbalance shifts endothelial cells toward a pro-inflammatory, pro-oxidant, and pro-apoptotic phenotype [3,6,15]. Under pathological stress, mitochondrial dysfunction may further increase ROS production, inflammatory activation, and cell death [15,16,18,35]. This mechanism strengthens the transition from metabolic stress to endothelial dysfunction within the gluco-vascular injury axis.

Mitochondrial injury also directly impairs the diabetic myocardium. Cardiomyocytes depend on mitochondrial ATP production for calcium handling, lipid metabolism, and contractile function [2,15,16]. Energetic deficiency can therefore lead to impaired calcium cycling, lipid accumulation, contractile dysfunction, cardiomyocyte apoptosis, fibrosis, and ventricular stiffness [2,15,16,19]. This provides a direct pathway from mitochondrial redox collapse to diabetic cardiomyopathy and heart failure.

**Figure 2:**
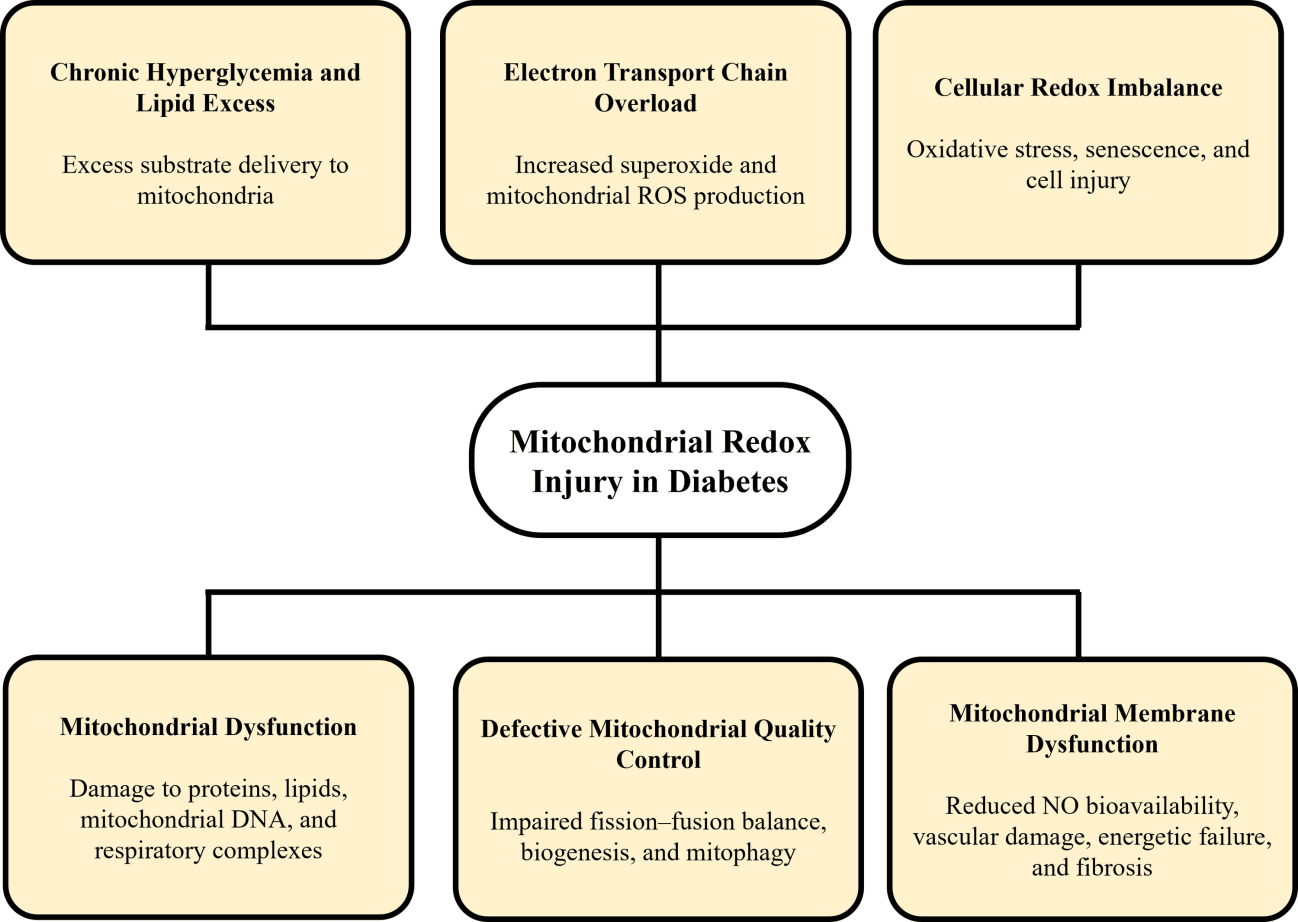
Mitochondrial Dysfunction and Redox Injury in the Gluco-Vascular Axis Created by the authors using Microsoft PowerPoint (Microsoft® Corp., Redmond, WA).

Therapeutically, mitochondrial dysfunction represents a rational target because it lies upstream of endothelial injury, oxidative vascular damage, and myocardial energetic failure. Potential strategies include antioxidants, AMPK activation, mitochondrial protective peptides, mitophagy modulation, improved metabolic efficiency, and oxidative stress reduction [2,15,16]. SGLT2 inhibitors and GLP-1 receptor agonists may also contribute to cardiovascular protection by improving substrate handling and reducing inflammation and mitochondrial stress [2,11,16]. The key takeaway is that mitochondrial redox collapse connects metabolic overload with endothelial dysfunction, microvascular injury, myocardial energetic failure, and progressive diabetic cardiovascular dysfunction [2,6,36]. The pathways of mitochondrial redox injury are shown in Figure 2.

Macrovascular injury: atherosclerosis, arterial stiffness, and ischemic disease

Macrovascular injury represents the large-vessel expression of the gluco-vascular injury axis [37]. In diabetes, endothelial dysfunction, oxidative stress, inflammation, lipid metabolism derangements, arterial stiffening, and vascular calcification converge to accelerate vascular disease [3,21-23,38]. These processes promote atherosclerotic plaque formation and increase the risk of ischemic events in the coronary, cerebral, and peripheral vascular beds [39]. Within the proposed axis, the importance of macrovascular injury lies not only in plaque formation or luminal obstruction, but also in how large-vessel disease amplifies downstream microvascular dysfunction and myocardial stress [40,41]. Defective nitric oxide signaling, oxidized lipoprotein accumulation, macrophage activation, vascular smooth muscle remodeling, and plaque vulnerability are key features of diabetic atherosclerosis [23,39]. Clinically, this helps explain why diabetic coronary artery disease is often diffuse, clinically silent, and complex [1,38].

Arterial stiffness links structural vascular remodeling with impaired cardiac loading conditions. Increased central arterial stiffness raises systolic load, reduces arterial compliance, impairs ventricular-vascular coupling, and can compromise coronary perfusion [38]. These changes may produce myocardial oxygen supply-demand mismatch, microvascular angina, silent ischemia, and adverse remodeling after ischemic injury [38]. In the gluco-vascular injury axis, arterial stiffness functions as a macro-to-microvascular amplifier: increased pulsatile pressure is transmitted into distal resistance vessels and capillary beds, worsening endothelial injury, capillary rarefaction, impaired vasodilatory reserve, and tissue hypoperfusion [38,41]. Peripheral artery disease is another manifestation of diabetic macrovascular injury and is characterized by atherosclerosis, endothelial dysfunction, reduced nitric oxide signaling, vascular stiffening, impaired angiogenesis, and progressive limb ischemia [39]. Thus, macrovascular disease can reduce conduit-vessel flow while simultaneously increasing small-vessel vulnerability, creating a dual burden of impaired delivery and impaired tissue-level perfusion [38,39,41].

Arterial stiffening is both a marker and a mechanism of diabetic macrovascular damage [38,41]. It is driven by collagen accumulation, elastin fragmentation, advanced glycation-related matrix crosslinking, vascular smooth muscle cell phenotypic switching, inflammation, oxidative stress, and medial calcification [29,38,40]. PWV reflects the cumulative burden of vascular ageing, metabolic injury, and structural remodeling [38,40]. Arterial stiffness is associated with increased ventricular afterload, left ventricular hypertrophy, impaired coronary perfusion, and higher heart failure risk [38]. Vascular calcification further worsens macrovascular disease by reducing arterial elasticity and increasing ischemic complications [40]. These changes interact directly with myocardial dysfunction by increasing afterload, reducing diastolic coronary perfusion, promoting ventricular-vascular uncoupling, and intensifying subendocardial ischemia [38,40]. In this way, large-vessel remodeling contributes to myocardial fibrosis and diastolic dysfunction even before overt ischemic events occur [2,38].

The macrovascular and microvascular components of diabetic vascular injury should not be interpreted separately. The association between diabetic retinopathy, arterial stiffness, and cardiovascular disease suggests shared mechanisms of vascular injury in type 2 diabetes [41]. The relationship between arterial stiffness and capillary abnormalities in systemic vascular disease also indicates that large-vessel stiffening may aggravate small-vessel damage [38,41]. This interaction is central to the proposed axis: macrovascular disease worsens myocardial outcomes through both direct hemodynamic load and indirect microvascular injury [38,41]. Atherosclerosis limits upstream flow, arterial stiffness increases pulsatile stress, and calcification reduces adaptive vascular compliance; together, these processes impair coronary microvascular reserve, promote hypoperfusion, increase myocardial oxygen demand, and contribute to fibrosis, diastolic dysfunction, and heart failure risk [8,38,42]. The key clinical takeaway is that diabetic macrovascular disease should be interpreted as part of a linked macrovascular-microvascular-myocardial continuum rather than as an isolated large-vessel complication. The main macrovascular mechanisms of diabetic cardiovascular dysfunction are listed in Table 3.

**Table 3:** Macrovascular Manifestations of the Gluco-Vascular Injury Axis NO: nitric oxide; PWV: pulse-wave velocity

Component	Main mechanism	Cardiovascular consequence	Reference
Macrovascular injury	Endothelial dysfunction, oxidative stress, inflammation, lipid dysregulation, arterial stiffness, and vascular calcification	Increased coronary, cerebral, and peripheral ischemic risk	[3,21-23,38]
Diabetic atherosclerosis	Impaired nitric oxide signaling, oxidized lipoprotein accumulation, macrophage activation, and smooth muscle remodeling	Plaque progression and plaque vulnerability	[23,39]
Nitric oxide depletion	Loss of vasodilatory, anti-inflammatory, and antithrombotic endothelial protection	Vasoconstriction, leukocyte adhesion, platelet activation, and plaque progression	[3,5,6,39]
Coronary artery disease	Diffuse coronary involvement with increased central arterial stiffness	Impaired coronary perfusion and myocardial oxygen imbalance	[1,38]
Arterial stiffness	Increased systolic load, reduced arterial compliance, and impaired ventricular-vascular coupling	Microvascular damage, tissue hypoperfusion, and heart failure risk	[38,41]
Peripheral artery disease	Atherosclerosis, endothelial dysfunction, reduced nitric oxide, vascular stiffening, and impaired angiogenesis	Progressive limb ischemia and higher ulceration risk	[39]
Vascular calcification	Medial stiffening, reduced vessel compliance, and altered blood-flow regulation	Worsened ischemic complications	[29,40]
Pulse-wave velocity	Marker of vascular aging, metabolic injury, and structural remodeling	Assessment of arterial stiffness burden	[38,40]
Shared macro-microvascular injury	Arterial stiffness associated with diabetic retinopathy and capillary abnormalities	Linked progression of macrovascular and microvascular damage	[41]

Microvascular injury and coronary microcirculatory dysfunction

Microvascular injury is the small-vessel expression of the gluco-vascular injury axis and provides a direct explanation for impaired myocardial perfusion in diabetes. It contributes to diabetic cardiovascular dysfunction by reducing tissue perfusion, oxygen delivery, and myocardial metabolic support [8,43-45]. Chronic diabetic metabolic stress promotes endothelial dysfunction, capillary basement membrane thickening, impaired angiogenesis, pericyte loss, and progressive microvascular rarefaction [8,43,44]. Microvascular rarefaction reduces capillary density and tissue perfusion reserve, thereby increasing susceptibility to hypoxia [43,44]. Capillary network loss also increases the diffusion distance between blood and cardiomyocytes, which may promote energetic stress, contractile dysfunction, and maladaptive myocardial remodeling [8,10,45]. This mechanism is clinically important because it can produce myocardial ischemia even in the absence of obstructive epicardial coronary artery disease [8,43,45].

Coronary microvascular dysfunction is characterized by impaired coronary flow reserve, coronary endothelial dysfunction, abnormal coronary vascular smooth muscle regulation, microvascular spasm, structural remodeling, and increased microvascular resistance [8,43-45]. Endothelial dysfunction reduces nitric oxide bioavailability and increases vasoconstrictive, inflammatory, and thrombotic signaling in the coronary microcirculation [3,6,8,44]. These changes disturb coronary autoregulation and reduce the ability of the microvasculature to match blood flow with myocardial oxygen demand [8,43-45]. Pericyte injury further destabilizes the capillary unit. Under normal conditions, pericytes regulate capillary tone, endothelial cell survival, capillary permeability, and basement membrane organization [43,44].

Loss or dysfunction of pericytes promotes capillary leakage, basement membrane thickening, impaired angiogenic repair, and microvascular dropout in diabetes [8,43,44]. These abnormalities may occur across several vascular beds and are relevant to coronary, retinal, renal, and peripheral microvascular complications [3,8,21,22,43]. Inflammation further worsens microvascular dysfunction by inducing leukocyte adhesion, endothelial cell swelling, glycocalyx degradation, platelet activation, and capillary plugging [3,6,8,13].

Additional microcirculatory insults, such as coronary microembolization, may aggravate perfusion heterogeneity and local inflammation. Coronary microembolization can impair vascular function and promote inflammation, edema, and heterogeneous myocardial perfusion [46]. These processes reduce myocardial blood flow and may cause repeated episodes of mild ischemia [43,45,46]. Over time, chronic microvascular inflammation and hypoperfusion promote cardiomyocyte stress, interstitial fibrosis, and impaired ventricular relaxation [8,10,45]. This explains the close relationship between microvascular injury and diabetic cardiomyopathy. Coronary microvascular dysfunction limits oxygen delivery, disrupts endothelial-cardiomyocyte communication, increases oxidative stress, and promotes myocardial fibrosis [8,10,46].

The key clinical takeaway is that microvascular dysfunction links systemic metabolic injury to cardiac organ dysfunction. It helps explain why patients with diabetes may develop heart failure, exercise intolerance, and diastolic dysfunction even without flow-limiting epicardial coronary stenosis [8,10,11,43]. Within the gluco-vascular injury axis, microvascular dysfunction is therefore the immediate vascular bridge between metabolic disease and myocardial failure.

Clinical translation, biomarkers, and therapeutic implications

Although the gluco-vascular injury axis remains a hypothesis-generating framework, it may have translational value by organizing therapeutic and diagnostic targets across the metabolic-vascular-myocardial continuum. Contemporary cardioprotective therapies in diabetes may act on several points within this axis. SGLT2 inhibitors and GLP-1 receptor agonists may provide cardiovascular benefit not only through glycemic effects, but also through improvements in substrate handling, inflammation, oxidative stress, endothelial function, vascular stiffness, renal-cardiac interaction, and heart failure risk [2,11,12,16]. These effects support the concept that cardiovascular protection in diabetes should extend beyond glucose-lowering alone.

Clinically applicable biomarkers may also help identify different stages of the proposed axis. Inflammatory markers such as high-sensitivity C-reactive protein and interleukin-6 may reflect inflammatory activation, while NT-proBNP and cardiac troponins may indicate myocardial stress or injury [11,13]. Endothelial dysfunction markers, arterial stiffness parameters such as PWV, coronary microvascular function indices, and imaging-based measures of myocardial structure and function may provide complementary information on vascular and myocardial involvement [7,8,38,41,43-45]. Echocardiography can support assessment of diastolic dysfunction and ventricular remodeling, whereas cardiac magnetic resonance imaging may help characterize myocardial fibrosis, deformation, and subclinical myocardial injury [2,10,11].

These tools should not be interpreted as a validated diagnostic panel for the gluco-vascular injury axis. Rather, they illustrate how the proposed framework could guide future prospective studies combining metabolic, endothelial, inflammatory, vascular, microvascular, and myocardial markers. At present, no biomarker panel, imaging strategy, or therapeutic algorithm has been validated specifically for this proposed axis. Validation studies are needed to determine whether such integrated biomarker and imaging strategies improve risk prediction, patient stratification, therapeutic selection, or cardiovascular outcomes beyond existing diabetic cardiomyopathy and heart failure models.

Limitations and future directions

This narrative review has a few limitations. First, the gluco-vascular injury axis is a conceptual framework derived from existing mechanistic, experimental, and clinical evidence, rather than a validated diagnostic or prognostic model. Its clinical utility remains uncertain because the framework has not yet been tested prospectively for risk prediction, patient stratification, therapeutic decision-making, or cardiovascular outcome prediction. At present, no evidence demonstrates that this framework improves clinical prediction, therapeutic decision-making, or cardiovascular outcomes compared with established diabetic cardiomyopathy, atherosclerosis, coronary microvascular dysfunction, or heart failure models. Second, the available evidence comes from heterogeneous experimental models, observational cohorts, and disease-specific studies, which limits direct comparison across diabetic populations. Third, endothelial dysfunction, oxidative stress, inflammation, microvascular impairment, and myocardial remodeling are assessed using different biomarkers and imaging methods, resulting in limited methodological uniformity. Fourth, many studies examine isolated metabolic, vascular, or cardiac pathways rather than evaluating the full metabolic-vascular-myocardial sequence.

The proposed axis should also be interpreted alongside complementary mechanisms of diabetic cardiovascular dysfunction, including autonomic dysfunction, neurohormonal activation, cardiorenal interactions, epicardial adipose tissue activity, and direct cardiomyocyte injury, which may modify or amplify the metabolic-vascular-myocardial sequence. Fifth, because this article is a narrative review, it did not use a formal systematic search strategy, predefined eligibility criteria, duplicate screening, risk-of-bias assessment, or meta-analytic methods. Therefore, selection bias cannot be excluded, and relevant studies may have been missed. These limitations should be considered when interpreting the axis as an integrative framework rather than a standardized clinical tool.

Future research should test whether this framework improves prediction, risk stratification, and treatment selection in diabetic cardiovascular disease. Large prospective studies should evaluate combined panels of metabolic, endothelial, inflammatory, imaging, microvascular, and cardiac biomarkers. Longitudinal studies are needed to determine whether early endothelial or microvascular dysfunction predicts later diabetic cardiomyopathy and heart failure. Multi-omics, single-cell, and spatial vascular biology approaches may help define tissue-level mechanisms linking metabolic overload to endothelial injury and myocardial remodeling. External validation studies are also needed to determine whether the proposed axis improves clinical interpretation beyond existing diabetic cardiomyopathy, atherosclerosis, and heart failure risk models. Clinical trials should examine whether interventions targeting glucose toxicity, endothelial injury, inflammation, oxidative stress, mitochondrial dysfunction, and microvascular impairment improve cardiovascular outcomes in diabetes.

The practical goal of future work should be to move the gluco-vascular injury axis from a narrative model toward a testable clinical framework for earlier detection and multi-targeted cardiovascular protection. Until such validation is available, the proposed axis should be interpreted as a hypothesis-generating framework rather than a validated clinical pathway.

## Conclusions

This narrative review presents the gluco-vascular injury axis as an integrated hypothesis-generating framework for understanding diabetic cardiovascular dysfunction. Cardiovascular injury in diabetes is not driven by chronic hyperglycemia alone. Insulin resistance, dyslipidemia, oxidative stress, inflammation, endothelial dysfunction, and microvascular damage also contribute to disease progression. Together, these mechanisms convert metabolic overload into vascular injury, which then promotes myocardial remodeling, fibrosis, diastolic dysfunction, and heart failure. Endothelial dysfunction is the central link in this sequence, connecting abnormal glucose and lipid metabolism with vascular inflammation, thrombosis, barrier disruption, and reduced tissue perfusion.

Macrovascular injury, arterial stiffness, coronary microvascular dysfunction, AGE-RAGE signaling, mitochondrial dysfunction, and redox imbalance further amplify this pathway. This interconnected model may help explain why diabetic cardiovascular disease can progress even when glucose control alone is addressed. The main clinical takeaway is that cardiovascular protection in diabetes may need to consider the full metabolic-vascular-myocardial pathway rather than glycemic control in isolation. A gluco-vascular perspective may support earlier detection of vascular injury, improved risk stratification, and broader therapeutic targeting, but its clinical value remains unproven. Prospective studies are needed to validate whether this framework improves prediction, patient stratification, therapeutic selection, or cardiovascular outcomes. Future prevention and treatment strategies should be evaluated to determine whether combining metabolic control with endothelial protection, anti-inflammatory approaches, mitochondrial support, microvascular preservation, and prevention of myocardial remodeling can reduce the cardiovascular burden of diabetes.
